# Supplementary material for: Lower plate serpentinite diapirism in the Calabrian Arc subduction complex
Source: Nat Commun. 2017 Dec 19;8:2172. doi: 10.1038/s41467-017-02273-x (PMC5736553; doi:10.1038/s41467-017-02273-x)
Supplement: Supplementary file 1 — Supplementary Information [file 41467_2017_2273_MOESM1_ESM.pdf]

a)

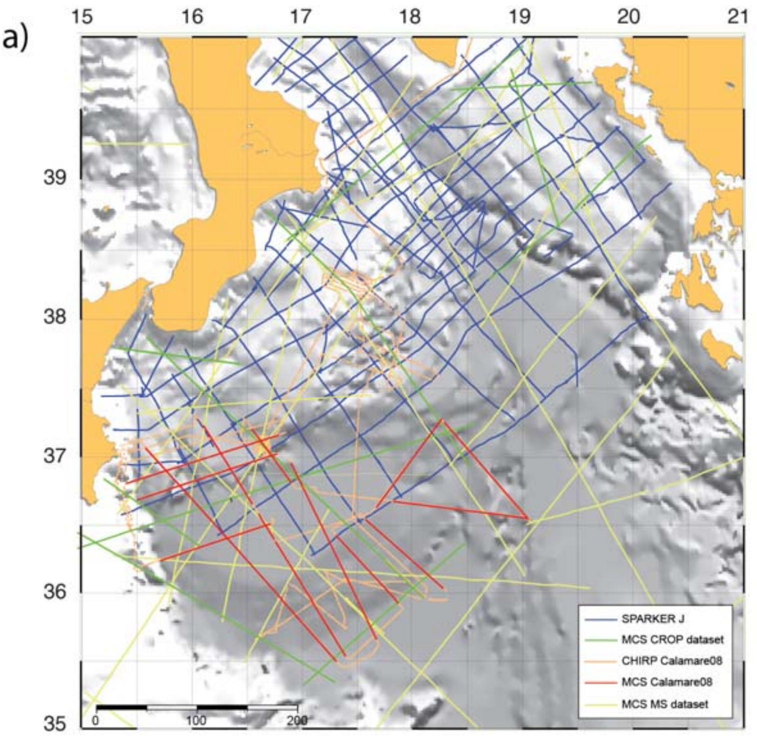

b)

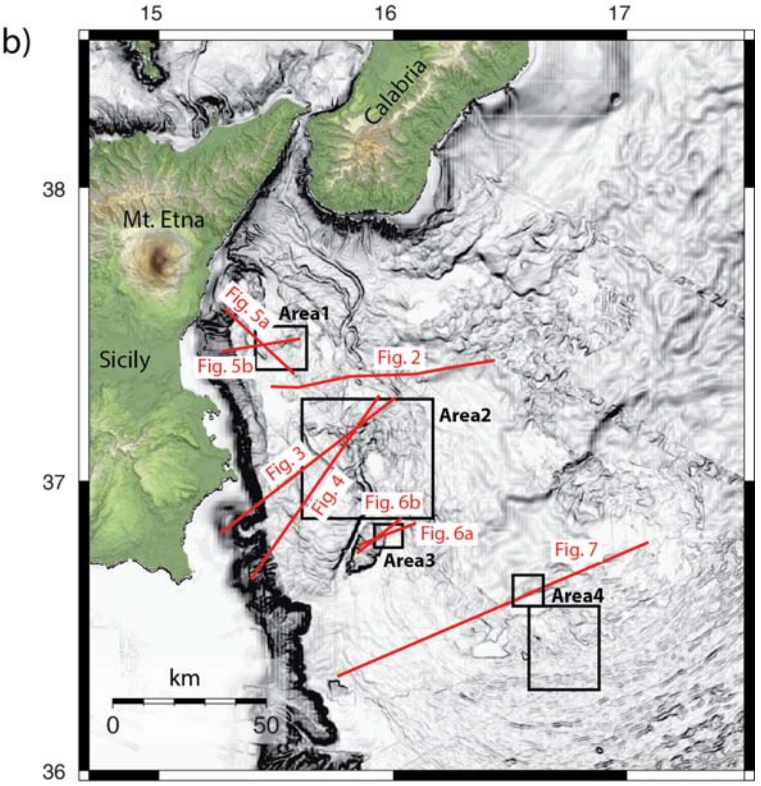

Supplementary Figure 1 – **Location of geophysical data**. a) Seismic reflection profiles used for structural interpretation; b) Location of seismic reflection profiles shown in Supplementary information.

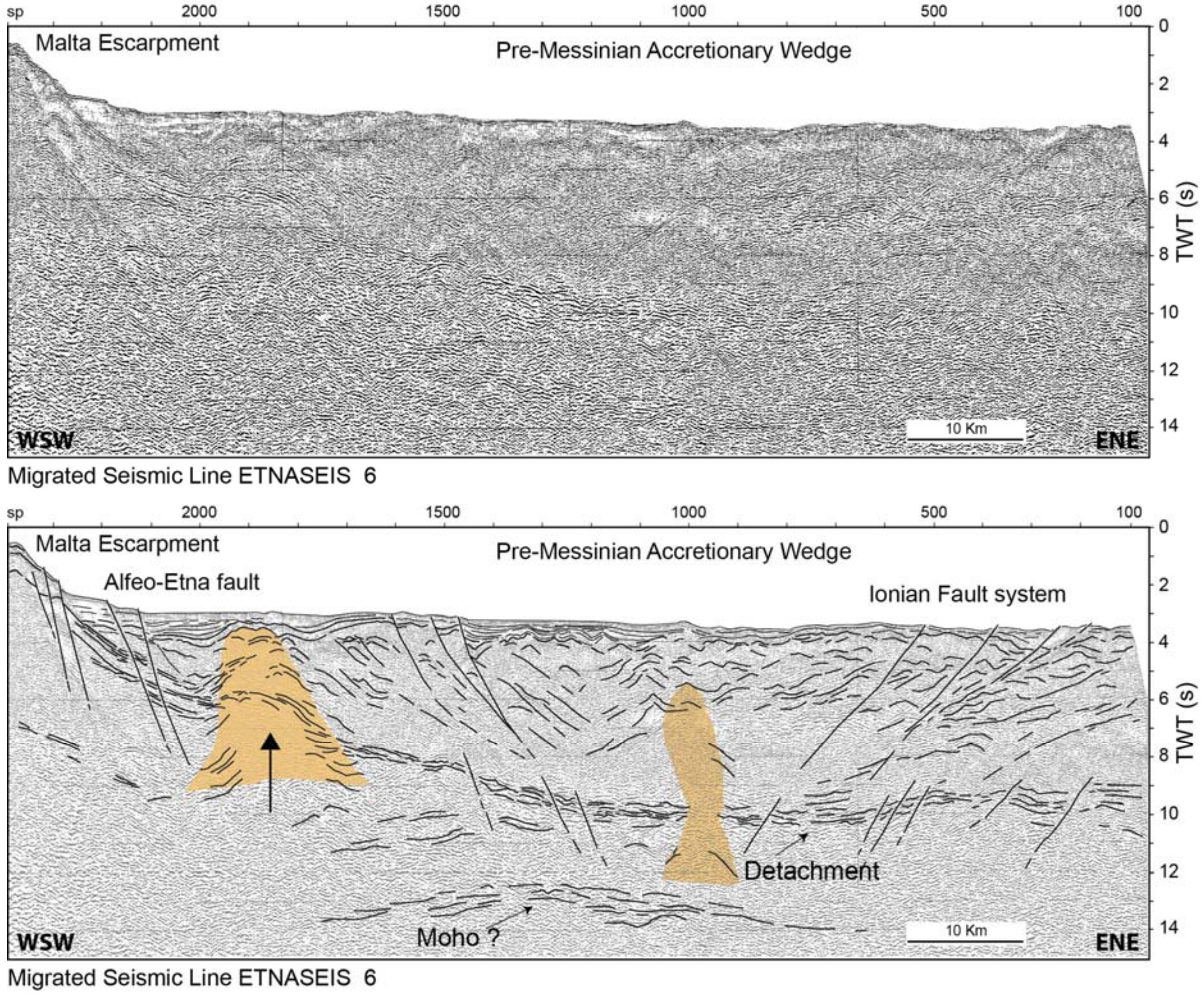

Supplementary Figure 2 – **Migrated multichannel seismic line ETNASEIS6**. This line is shown in the manuscript in Fig. 3a. Un-interpreted seismic line (above) and its line drawing (below). Seismic line location in Fig. 1. Yellow/brown pattern: diapiric features.

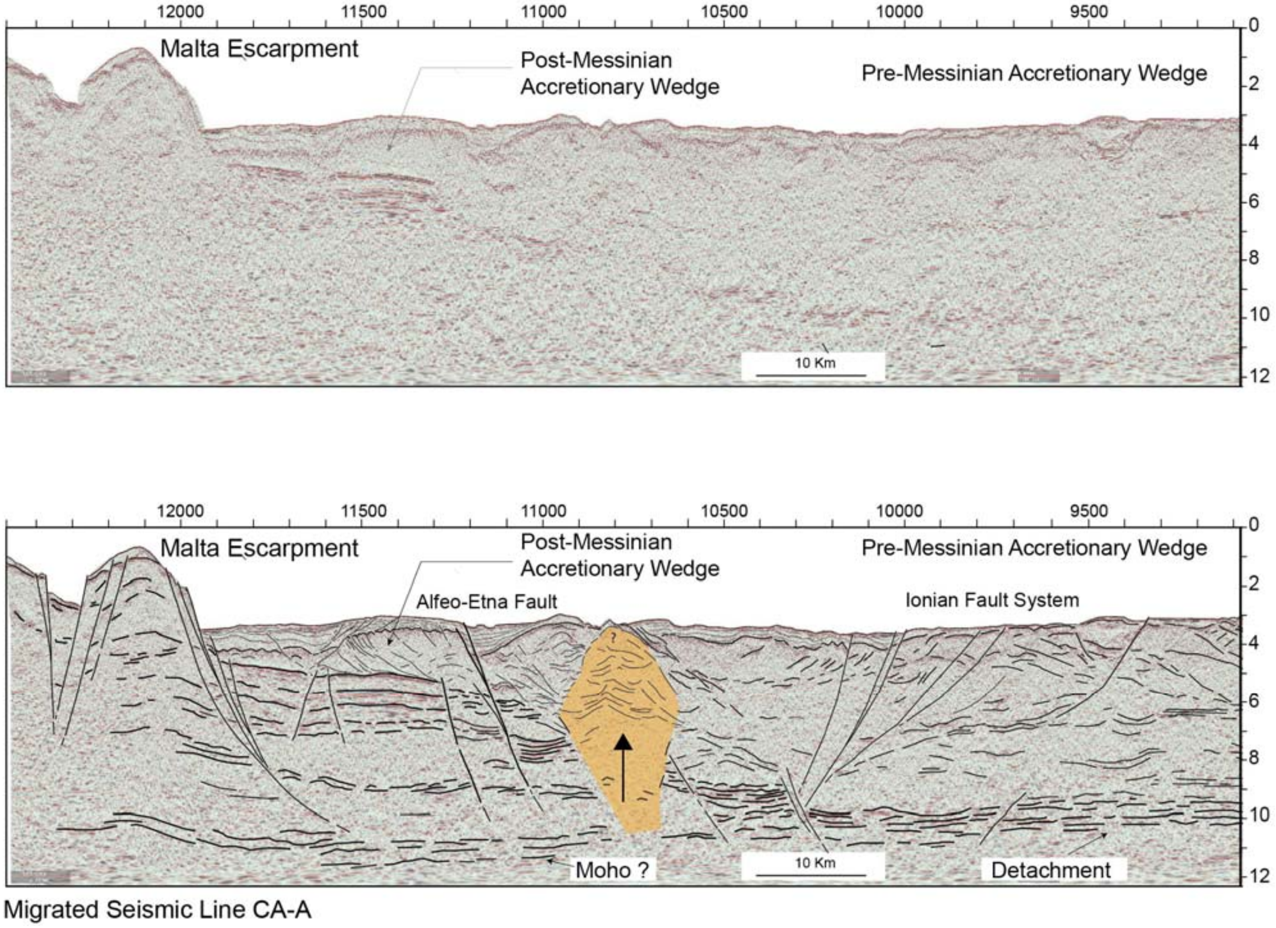

Supplementary Figure 3 – **Migrated multichannel seismic line CA-A**. This line is shown in the manuscript in Fig. 3b. Un-interpreted seismic line (above) and its line drawing (below). Seismic line location in Fig. 1. Yellow/brown pattern: diapiric features.

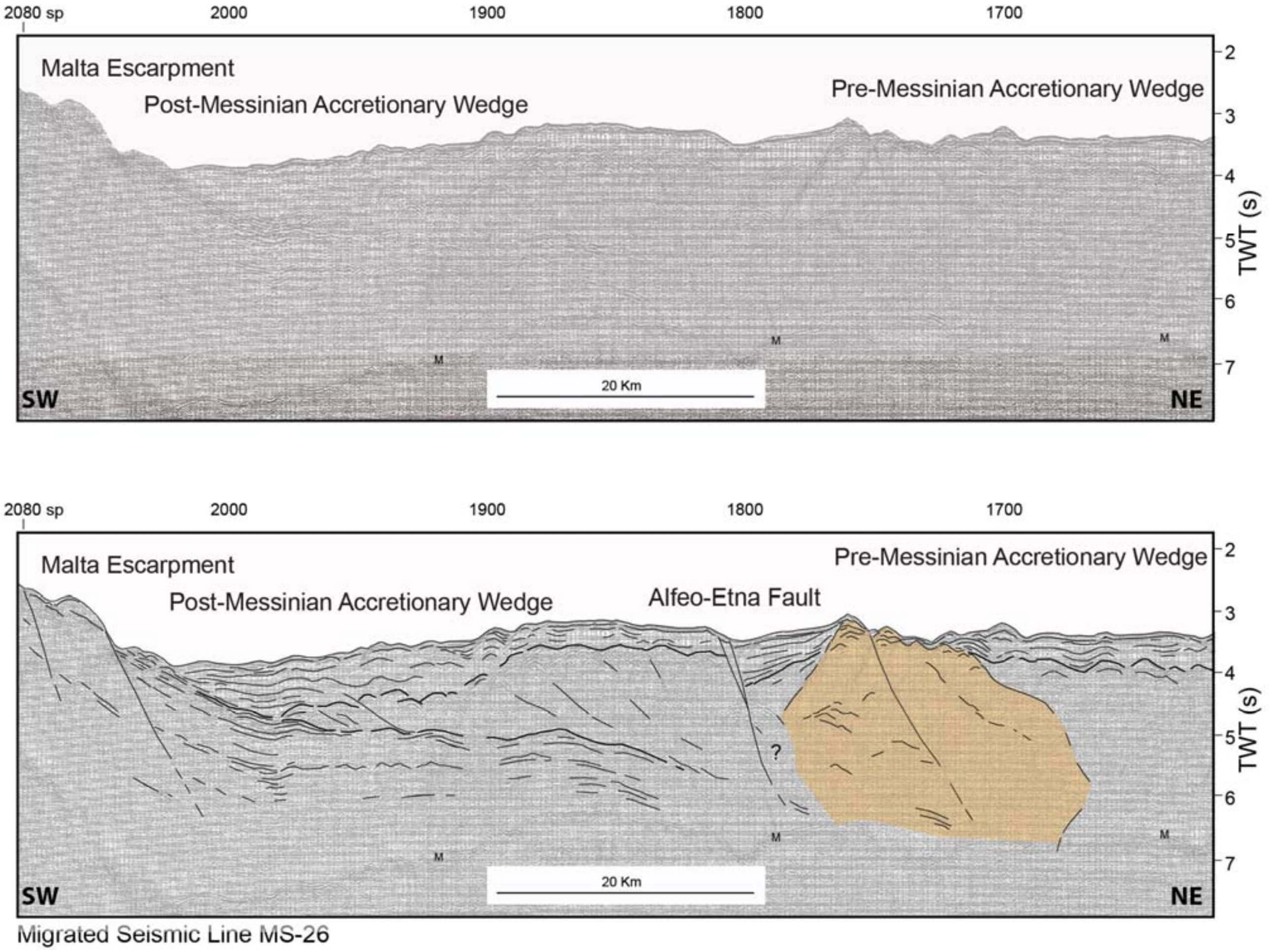

Supplementary Figure 4 – **Migrated multichannel seismic line MS-26**. This line is shown in the manuscript in Fig. 3c. Un-interpreted seismic line (above) and its line drawing (below). Seismic line location in Fig. 1. Yellow/brown pattern: diapiric features.

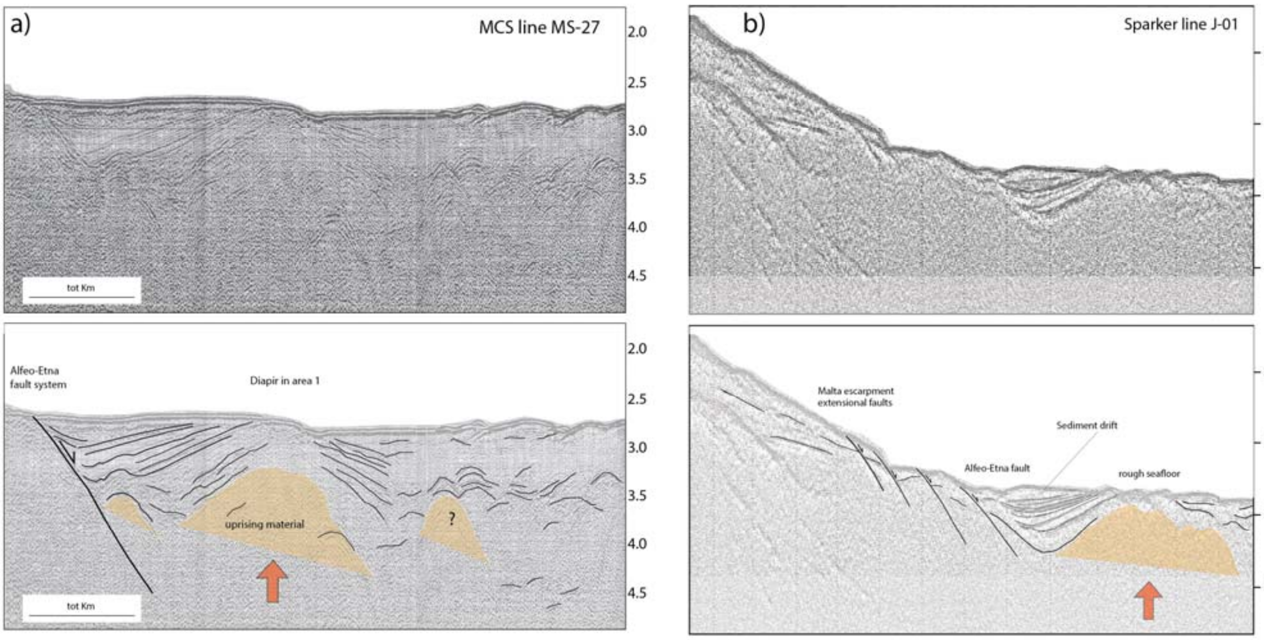

Supplementary Figure 5 – **Seismic reflection lines in area 1.** a: Migrated MCS line MS-27. b: Sparker line J-01. Seismic line location in Fig. 1. Yellow/brown pattern: diapiric features.

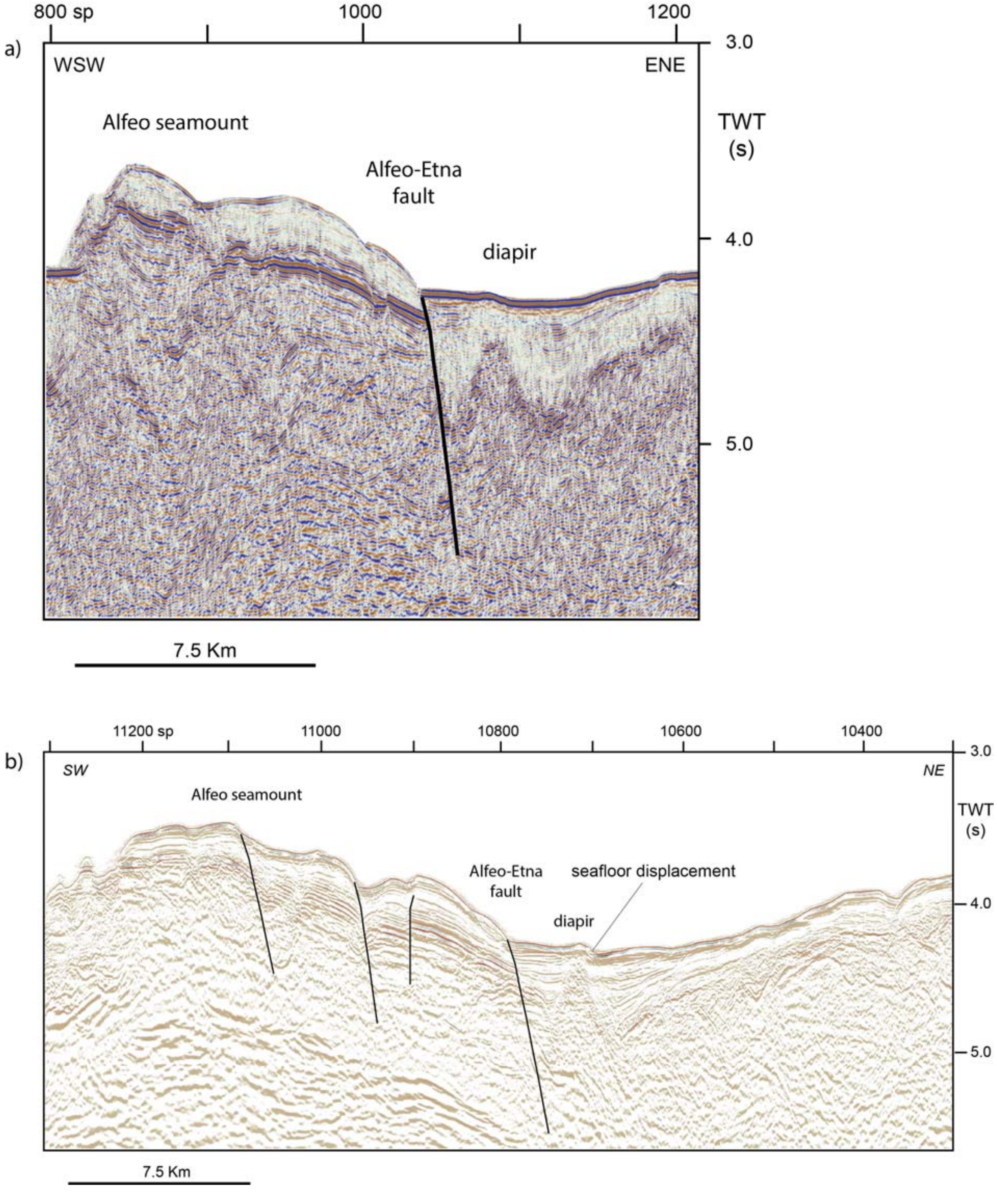

Supplementary Figure 6 - Seismic reflection lines in area 3. a: Migrated MCS line CALA-02. b: Migrated MCS line CA-B. Seismic lines location in Fig. 1.

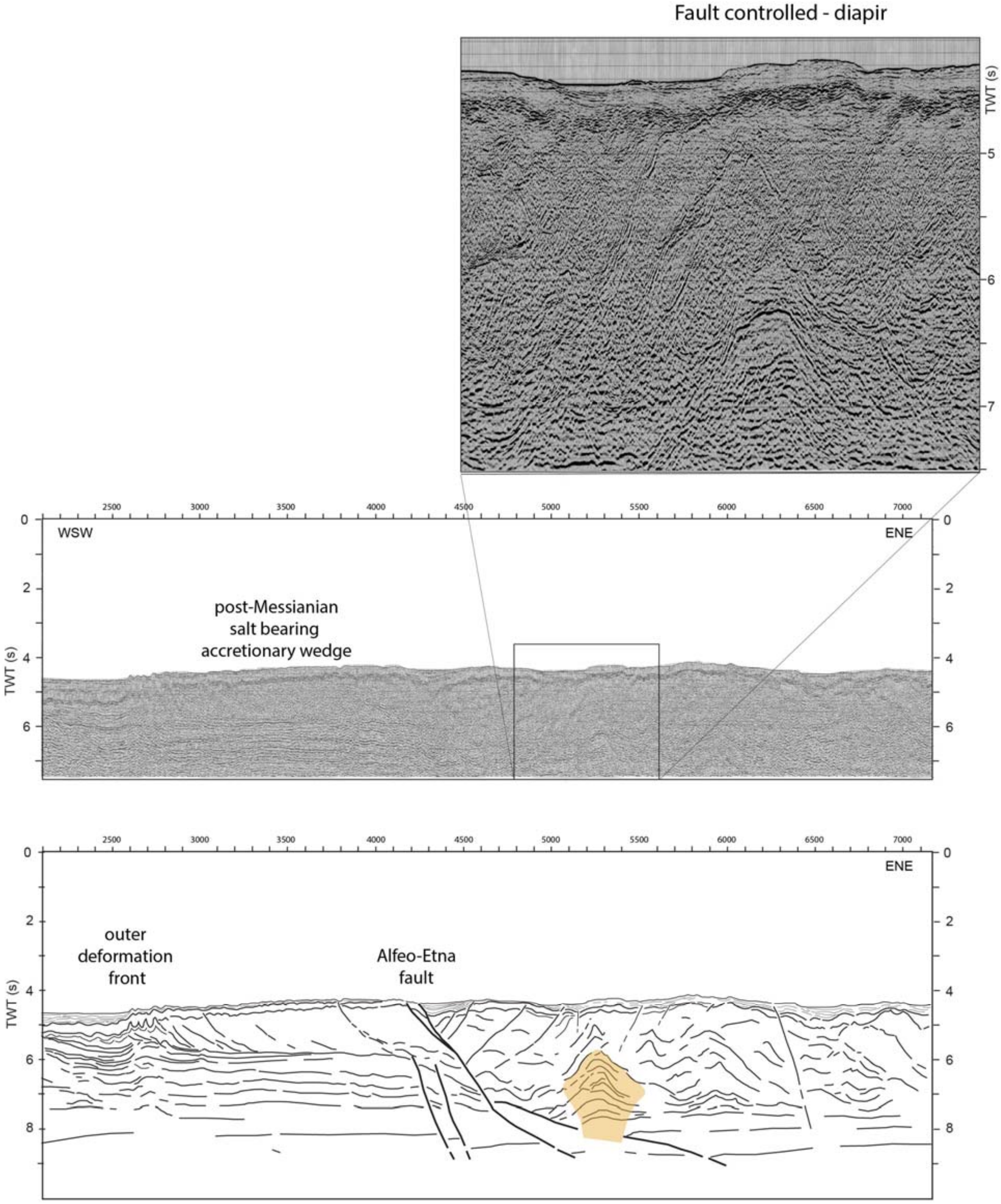

Supplementary Figure 7 - Migrated multichannel seismic line CA-C in area 4. Un-interpreted seismic line (above) and its line drawing (below). The line drawing below 7 sec TWT was drawn thanks to correlation with other deep penetration seismic lines. The inset highlights the geometry of the fault-controlled diapiric feature. Seismic line location in Fig. 1. Yellow/brown pattern: diapiric features.

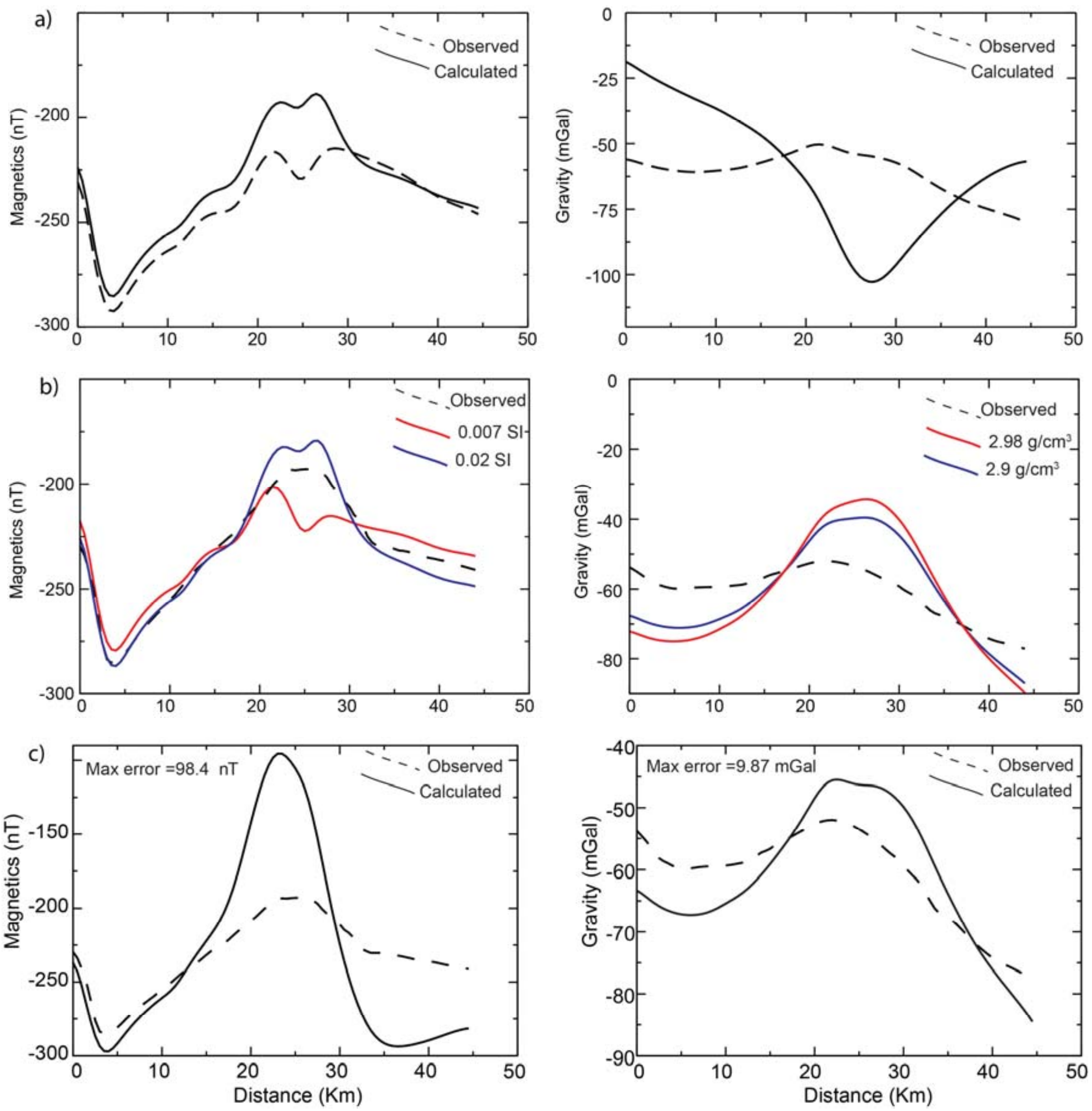

Supplementary Figure 8. Gravity and magnetic models with different diapirs lithologies. a) Mud diapir (0.0 SI and 2.2 gcm^{-3}). Modelled gravity profile does not match the observed signal with a maximum misfit error of about 47.1 mGal. If we assume a lower density of 1.75 to 1.92 g cm^{-3} as in mud volcanoes of the Mediterranean ridge [1] the misfit error of the modelled gravity anomaly increases; b) Gabbroic cumulates with susceptibility values taken from [2] (red line) and [3] (blue line). The density of gabbros is well constrained in the range of 2.9 g cm^{-3} [4,5] up to 2.98 g cm^{-3} [6]. Such high density values for the intrusive body in our modelling results in a gravity anomaly not fitting the acquired data, producing a maximum discrepancy larger than 15 mGal, suggesting that the hypothesis of an intrusive gabbroid nature is unlikely for the Ionian Sea diapirs. c) Basaltic dykes: we used a classic sequence of oceanic crust in a typical low spreading center[7,8] where low serpentinized mantle peridotites (0.001 SI and 3.0 g/cm^3) are covered by gabbros (0.0017 SI and 2.95 g cm^{-3}) and basaltic dikes swarm (0.005 SI and 2.85 g cm^{-3}). Both magnetic and gravity calculated profiles differ from those observed with a maximum misfit error of 9.8 mGal and 98 nT, respectively. The hypothesis of an intrusive ensemble made of gabbro and basaltic dikes appears to be unlikely.

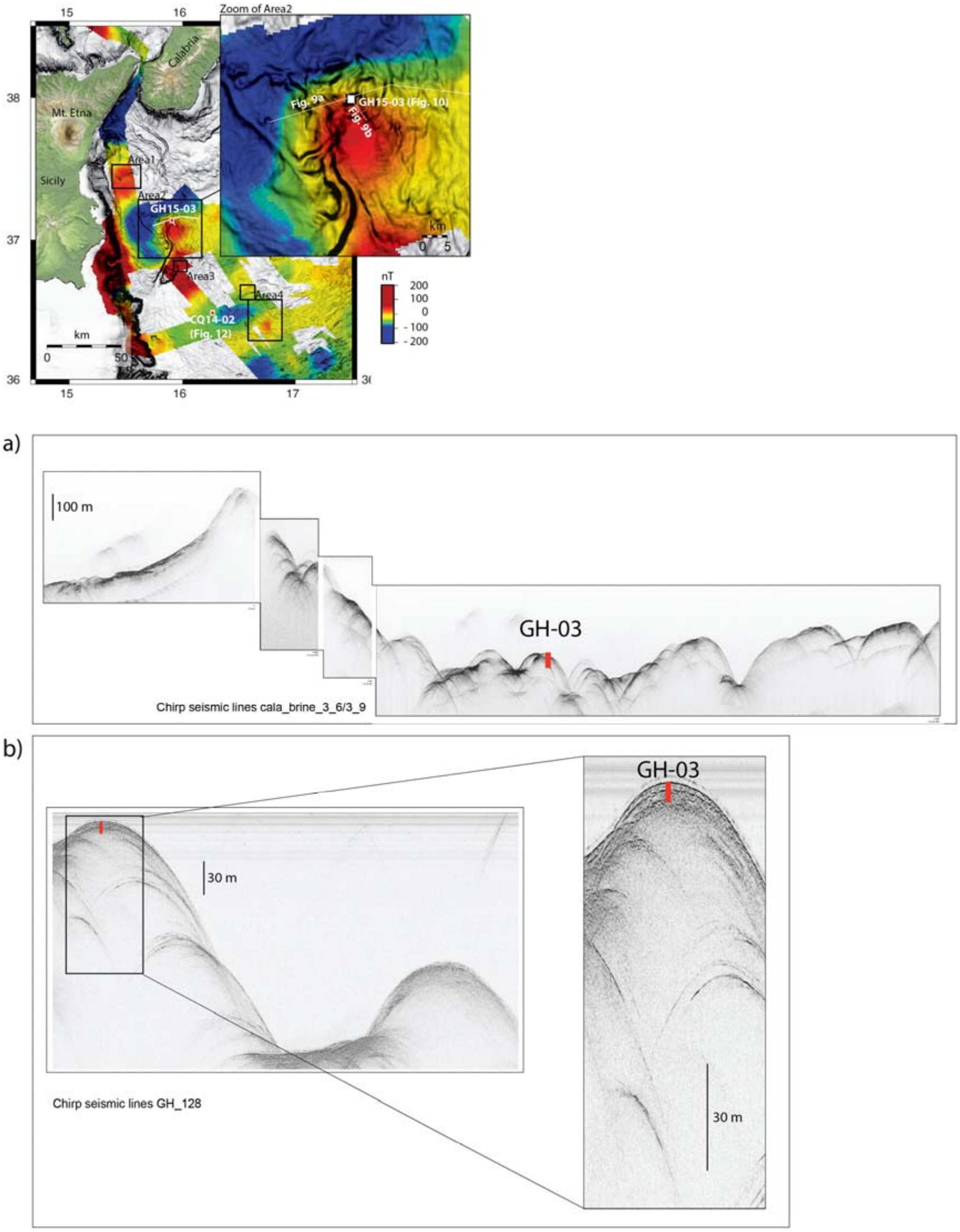

Supplementary Figure 9 - Location of sediment cores. Core GH15-03 was collected on top of the diapir in area 2, while core CQ14-02 in a region not affected by diapirism (see red squares in the multibeam map with superposed magnetic gravity anomalies). The 5.5 m long sediment core GH15-3 (core b in the manuscript), was recovered at about 2916 m water depth. The coring station is located on top of a structural high, identified by seismic reflection data. The top of this diapiric structure is close to but does not reach the seafloor. a) and b) Chirp profiles collected during coring operations within the rough seafloor morphology corresponding to the Area 2 described in the manuscript. Chirp lines location in the multibeam map.

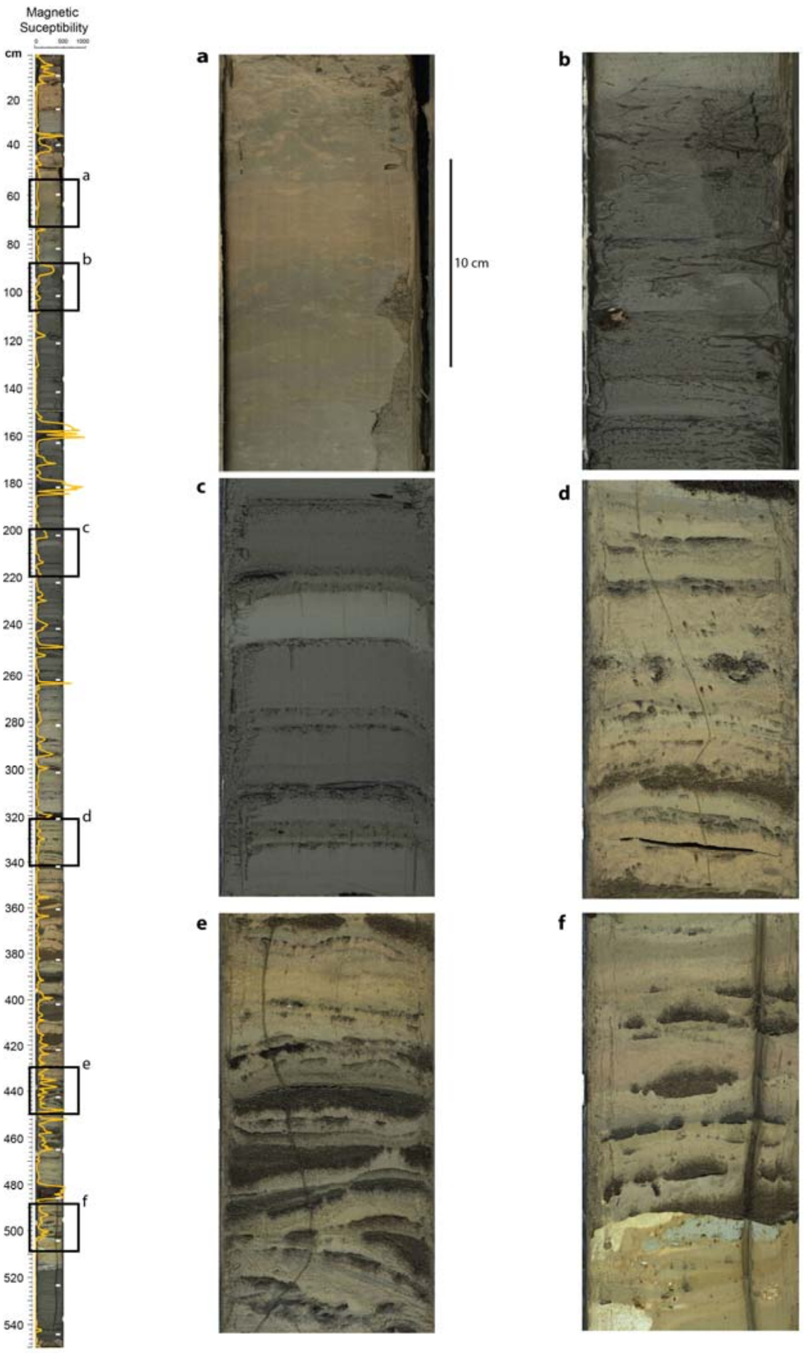

Supplementary Figure 10 – Sediment core GH15-03. Left side: core photograph and overprinted magnetic susceptibility in yellow. White boxes represent PW samples described in the manuscript. Right side: zoom of core sections. The recentmost core sections (a, b, c) show undeformed, parallel-layered sediment beds. The lower sections exhibit strong sediment reworking and disturbances, layer discontinuity, patchy cloudy and consolidated facies[9], and indications for vertical fluid conduits and mud injections (d, e, f).

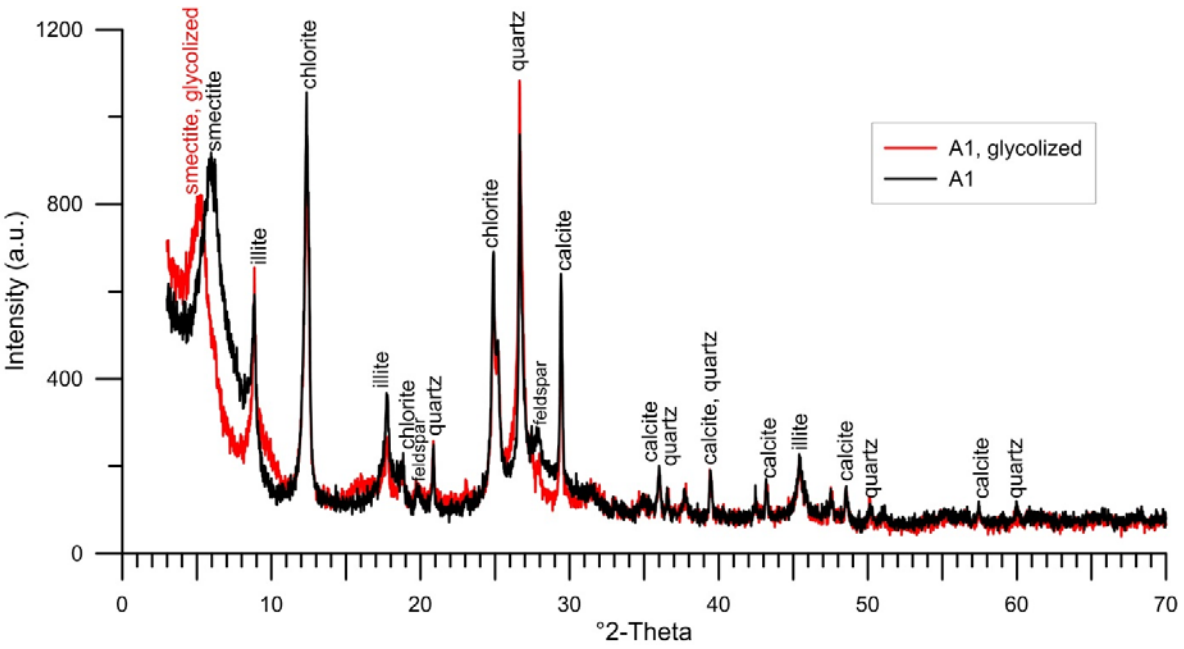

Supplementary Figure 11 - X-ray diffraction analysis of core GH15-03. Bulk sediment sample A1 has been subsampled at the base of core GH15-03 (core photography in Supplementary Figure 10) between 529 and 530 cm. Main components are smectite (17 %), illite (24 %), chlorite (29 %), quartz (9 %), feldspar (10 %) and calcite (11 %). Comparison of glycolized and non-glycolized diffraction patterns confirms the occurrence of smectite and possibly of a mixed illite/smectite layer phase [10]. Interestingly a chlorite, i.e. the Mg-rich clinochlore, was identified as the main component with (up to 29 wt.%) in sample A1. Chlorite is often associated with serpentine and even builds mixed-layered serpentine/chlorites[10]. However, serpentine minerals (lizardite, chrisotile, antigorite) were not clearly identified in A1 diffraction pattern when using procedures described by[10]. Identified chlorites could be formed by sub-sedimentary metamorphic rock alteration and uplifted by diapiric transport or formed by authigenic precipitation e.g. at subsea hydrothermal vent areas[11]. Moreover, considering large terrestrial metamorphic rock and serpentine deposits in the region (e.g.[12,13]) a detrital source of these clay minerals cannot be excluded.

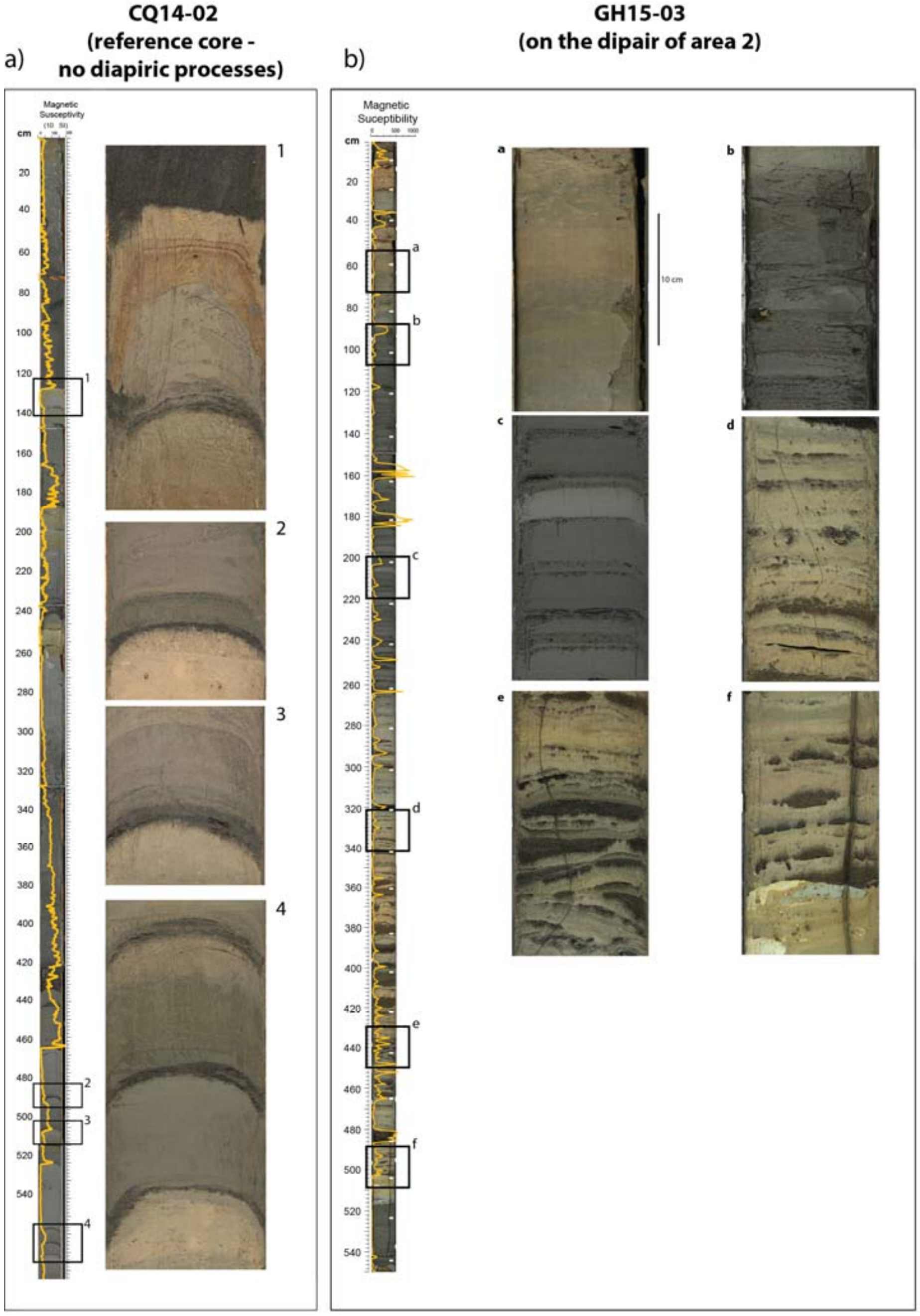

Supplementary Figure 12 – Comparison between sediments above the diapir (core b in the manuscript) and a reference core in a region not affected by diapirism (core d in the manuscript). a) The reference core CQ14-02 is characterized by alternation of pelagic sediments and turbidite beds. Sediment layers appear to be undisturbed and continuous (insets 2, 3, 4) and the uppermost muddy units of the turbidites show evidence of undisturbed planar millimetric laminations not affected by sediment disruption (inset 1). b) Core GH15-03, collected in the diapiric field of area 2, shows clear evidence of sediment layering disruption increasing towards the base of the core (Supplementary Fig. 10).

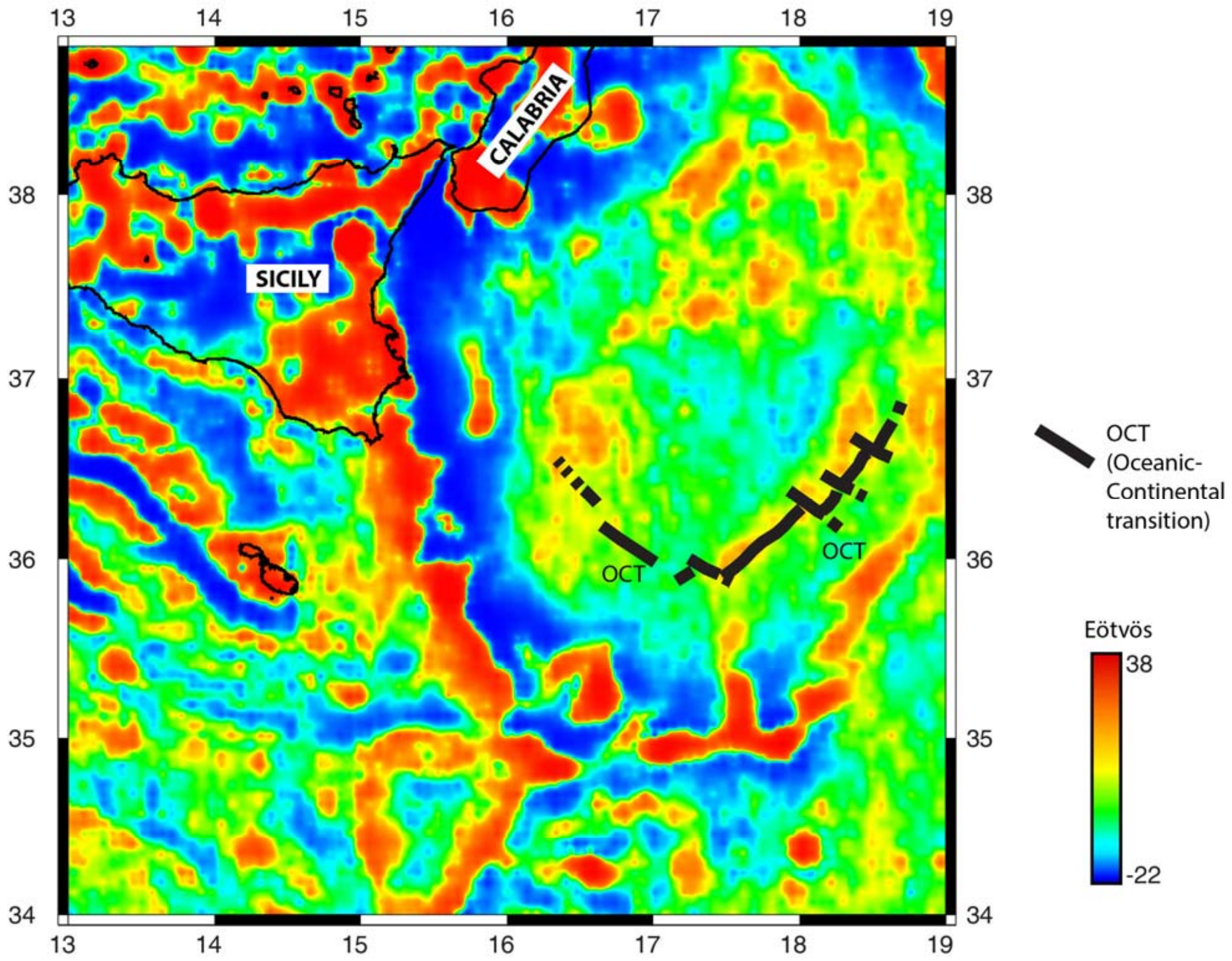

Supplementary Figure 13 - Gravity data and OCT geometry. Distribution of Vertical Gravity Gradient derived from satellite free air gravity data obtained during the recent CryoSat-2 and Jason-1 altimeter mission (ver. 23.1 [14]). VGG map in the Ionian Sea shows clearly the western boundary of the subducting plate marked by a set of vertical displacements along the Malta escarpment. Very peculiar low amplitude VGG variations not correlated with seafloor morphology are also observed within the basin recalling the transition between oceanic and continental domains as observed in the westernmost Herodotus basin[15]. Gravity map was generated using the GMT software [16].

Supplementary References

1. Camerlenghi, A. *et al.* Geophysical evidence of mud diapirism on the Mediterranean Ridge accretionary complex. *Mar. Geophys. Res.* **17,** 115–141 (1995).

2. Dick, H. J. B., Natland, J. H. & Miller, D. J. *Proceedings of the Ocean Drilling Program, 176 Initial Reports*. **176,** (Ocean Drilling Program, 1999).

3. Richter, C., Kelso, P. R. & MacLeod, C. J. Magnetic Fabrics and Sources of Magnetic Susceptibility in Lower Crustal and Upper Mantle Rocks from Hess Deep. in *Proceedings of the Ocean Drilling Program, 147 Scientific Results* (Ocean Drilling Program, 1996). doi:10.2973/odp.proc.sr.147.025.1996

4. Hyndman, R. D. & Drury, M. J. The physical properties of oceanic basement rocks from deep drilling on the Mid-Atlantic Ridge. *J. Geophys. Res.* **81,** 4042–4052 (1976).

5. Manea, M. & Manea, V. C. On the origin of El Chichón volcano and subduction of Tehuantepec Ridge: A geodynamical perspective. *J. Volcanol. Geotherm. Res.* **175,** 459–471 (2008).

6. Carlson, R. L. & Raskin, G. S. Density of the ocean crust. *Nature* **311,** 555–558 (1984).

7. Gee, J. S. & Kent, D. V. Source of Oceanic Magnetic Anomalies and the Geomagnetic Polarity Timescale. in *Treatise on Geophysics* **5,** 455–507 (Elsevier, 2007).

8. Dunn, R. A. & Forsyth, D. W. Crust and Lithospheric Structure – Seismic Structure of Mid-Ocean Ridges. in *Treatise on Geophysics* 419–443 (Elsevier, 2007). doi:10.1016/B978-044452748-6.00012-2

9. Panieri, G. *et al.* Mud volcanoes along the inner deformation front of the Calabrian Arc accretionary wedge (Ionian Sea). *Mar. Geol.* **336,** 84–98 (2013).

10. Moore, D. M. & Reynolds, R. C. *X-ray diffraction and identification and analysis of clay minerals.* (Oxford University Press, 1989).

11. Lackschewitz, K. S. *et al.* Mineralogical, geochemical and isotopic characteristics of hydrothermal alteration processes in the active, submarine, felsic-hosted PACMANUS field, Manus Basin, Papua New Guinea. *Geochim. Cosmochim. Acta* **68,** 4405–4427 (2004).

12. Manuella, F. C., Carbone, S. & Barreca, G. Origin of Saponite-Rich Clays in a Fossil Serpentinite-Hosted Hydrothermal System in the Crustal Basement of the Hyblean Plateau (Sicily, Italy). *Clays Clay Miner.* **60,** 18–31 (2012).

13. Barreca, G. Geological and geophysical evidences for mud diapirism in south-eastern Sicily (Italy) and geodynamic implications. *J. Geodyn.* **82,** 168–177 (2014).

14. Sandwell, D. T., Muller, R. D., Smith, W. H. F., Garcia, E. & Francis, R. New global marine gravity model from CryoSat-2 and Jason-1 reveals buried tectonic structure. *Science (80-.).* **346,** 65–67 (2014).

15. Granot, R. Palaeozoic oceanic crust preserved beneath the eastern Mediterranean. *Nat. Geosci.* **9,** 701–705 (2016).

16. Wessel, P. & Smith, W. H. F. New, improved version of generic mapping tools released. *Eos, Trans. Am. Geophys. Union* **79,** 579–579 (1998).
